# Supplementary material for: Self-sustained electricity generator driven by the compatible integration of ambient moisture adsorption and evaporation
Source: Nat Commun. 2022 Jun 25;13:3643. doi: 10.1038/s41467-022-31221-7 (PMC9233698; doi:10.1038/s41467-022-31221-7)
Supplement: Supplementary file 1 — Supplementary Information [file 41467_2022_31221_MOESM1_ESM.pdf]

Self-sustained electricity generator driven by the compatible integration of ambient moisture adsorption and evaporation

Jin Tan[1], Sunmiao Fang[1], Zhuhua Zhang[1,2], Jun Yin[1], Luxian Li[1], Xiang Wang[1], Wanlin Guo[1,2]*

[1]Key Laboratory for Intelligent Nano Materials and Devices of the Ministry of Education, State Key Laboratory of Mechanics and Control of Mechanical Structures, Nanjing University of Aeronautics and Astronautics, Nanjing, 210016, China.

[2]Institute for Frontier Science, Nanjing University of Aeronautics and Astronautics, Nanjing, 210016, China.

***Corresponding author.** E-mail: wlguo@nuaa.edu.cn

This file includes:

Supplementary Figures 1-25

Supplementary Notes 1-7

Supplementary Movies 1-3

Supplementary References

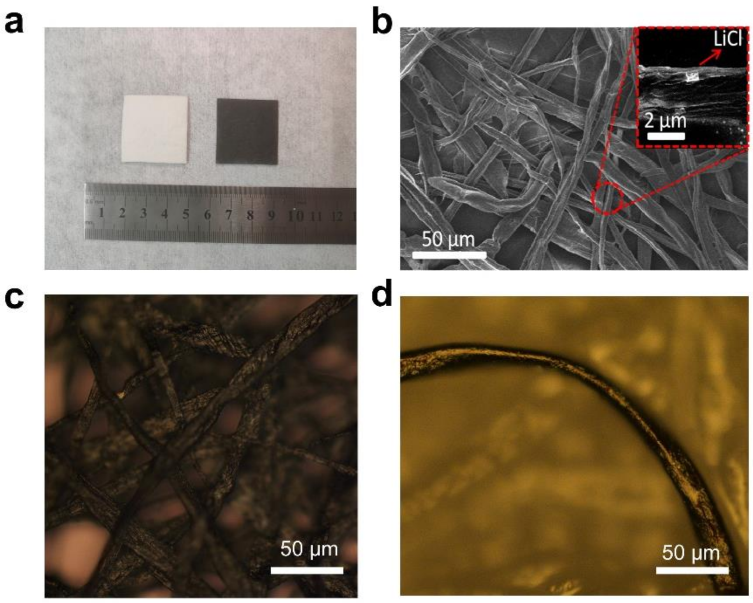

Supplementary Fig. 1 | Morphologies of the hygroscopic layer and the evaporative layer. (a) Photograph of the hygroscopic layer and evaporative layer composed of LiCl-loaded and carbon-black-loaded cellulon paper slice. (b) Scanning electron microscope (SEM) image and its partial magnification (inset) of the LiCl-loaded cellulon paper slice. Microphotographs taken from (c) the evaporative layer and (d) a single carbon-black-loaded fiber.

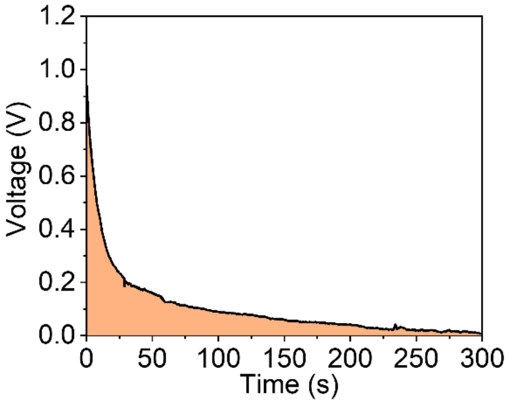

Supplementary Fig. 2 | Galvanostatic discharge curve of the SSEG at 25 °C, 60% RH. The discharge current is 9 μA.

Supplementary Note 1.

The energy density of the generators was measured via galvanostatic discharge method and further calculated as:

$$E = I \times \int_0^t U,$$

where *I, U, t* are the discharge current, voltage and discharge time, respectively. The gravimetric energy density (E_m) and volumetric energy density (E_v) were calculated as:

$$E_m = \frac{E}{m},$$

$$E_v = \frac{E}{Ad},$$

where *m, A, d* are the mass, area and thickness of bilayer heterogenous materials, respectively. A gravimetric energy density of 0.15 mW·h·kg^{-1} and a volumetric energy density of 0.04 mW·h·L^{-1} are obtained at 25 °C, 60% RH.

The maximum power density of the device at different humidity was calculated as:

$$P_{max} = V \times J,$$

where *V* and *J* are the open-circuit voltage and the short-circuit current density, respectively. The areal power density approaches 32.9 nW cm^{-2} and 0.7 μW cm^{-2} at 20% and 60% RH (25°C), respectively.

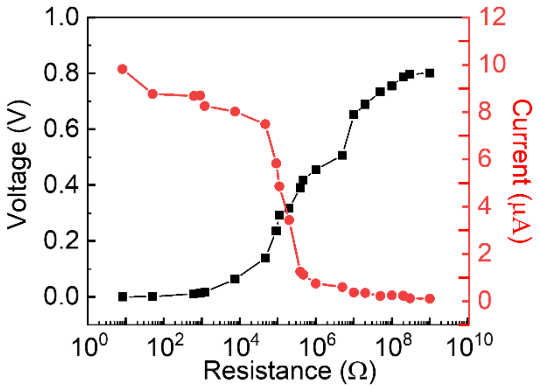

Supplementary Fig. 3 | Electric outputs from the SSEG at different load resistances (R_L). The testing was performed in the ambient environment at 55% RH.

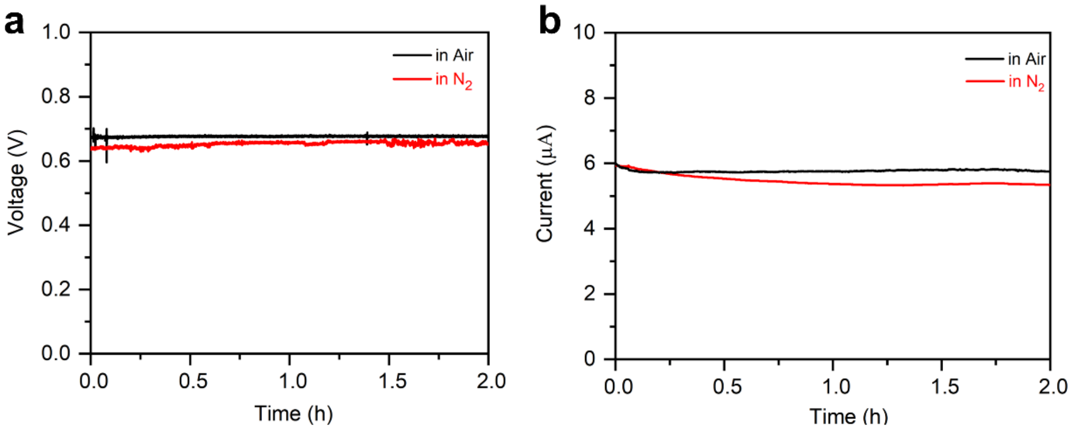

Supplementary Fig. 4 | Electric outputs of the SSEG in different atmosphere. (a) The open-circuit voltage and (b) The short-circuit current delivered by a device exposed to air and nitrogen. The temperature and RH were maintained at 22-25 ℃, 60-70%.

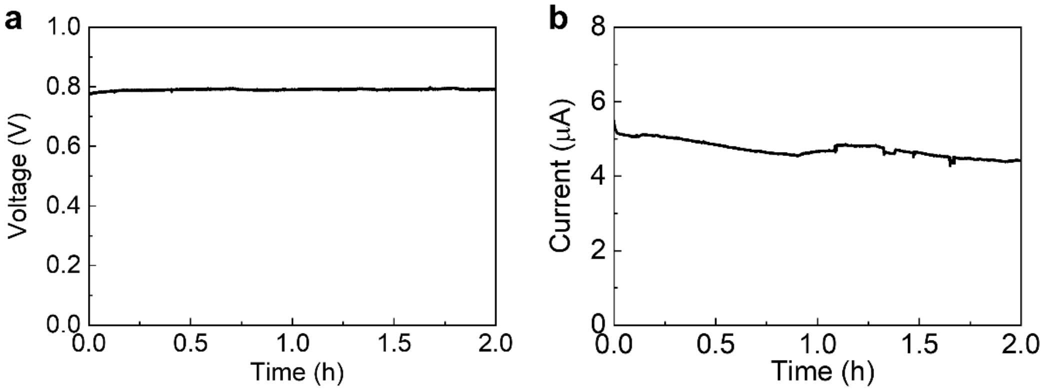

Supplementary Fig. 5 | Electric outputs of the SSEG with gold electrodes. (a) The open-circuit voltage and (b) The short-circuit current delivered by a device using a pair of gold electrodes. Both of these two signals were measured in an ambient environment of 25 ℃, 60% RH.

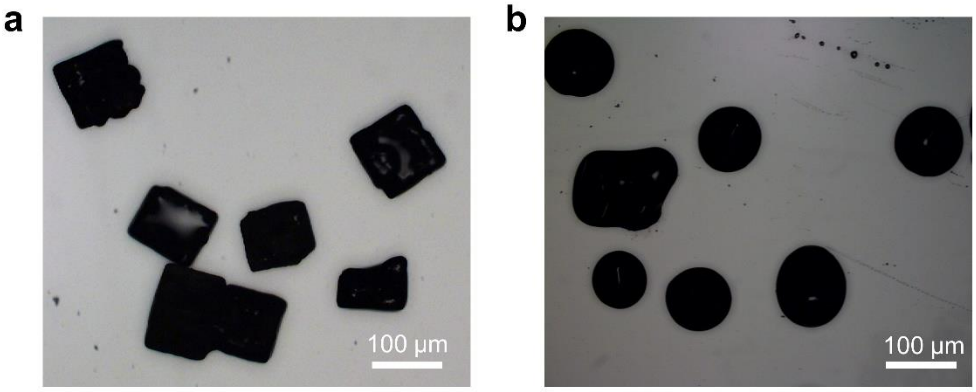

Supplementary Fig. 6 | Deliquescence of lithium chloride in air. Microscopic images of lithium chloride exposed to 40% RH for (a) 1 minute and (b) 15 minutes.

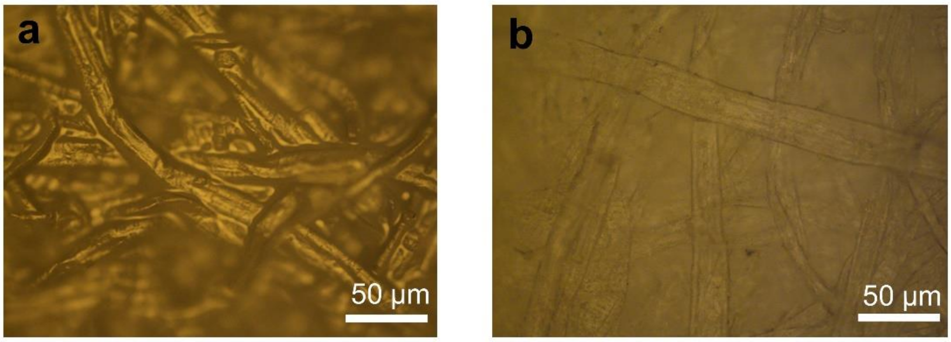

Supplementary Fig. 7 | Hydrophilicity and porous nanofibrous structure of the cellulon paper. Microphotographs taken from (a) the outside surface and (b) internal surface of the bilayer fibrous structure with thickness of 4mm. The hygroscopic was fabricated by steeping cellulon paper slice into 30 wt % LiCl solution and drying. After being exposed to atmosphere for 30min, the bilayer fibrous structure was transferred into a refrigerator to fix the water before observation.

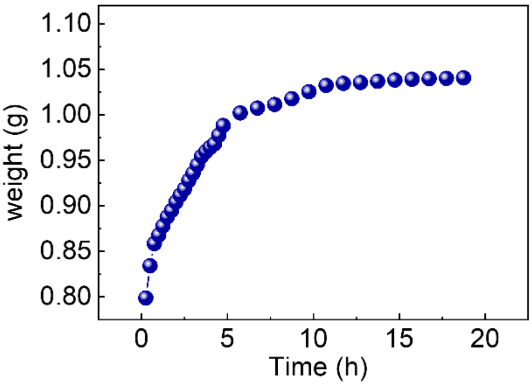

Supplementary Fig. 8 | Evolution of SSEG weight during the device operation at 25 ℃, 70% RH.

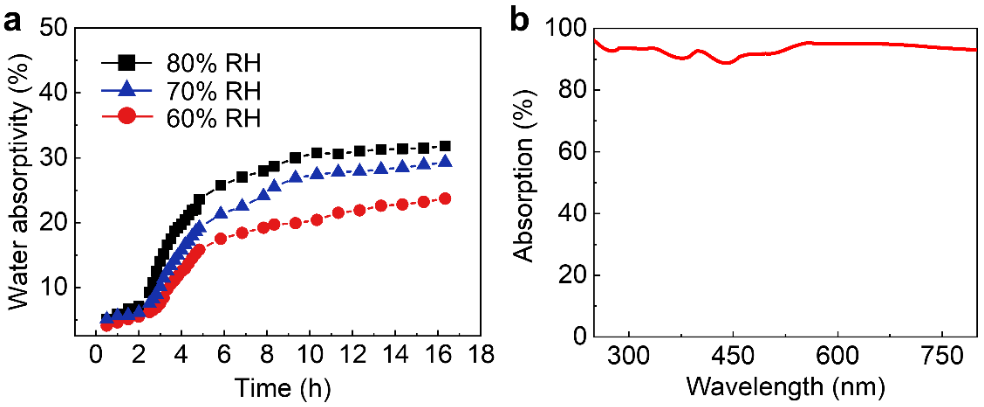

Supplementary Fig. 9 | Water absorptivity and light absorptivity of the evaporative layer. (a) Water absorptivity of the evaporative layer at 25 ℃ and different RH. (b) Experimental absorption spectra of the evaporative layer measured in the range of 250-800 nm.

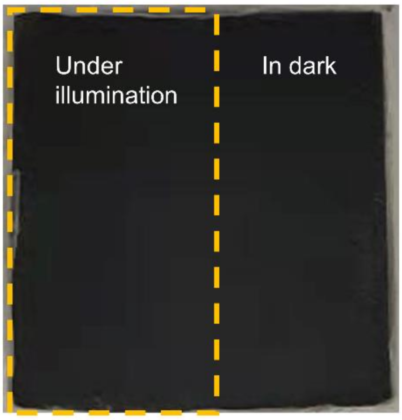

Supplementary Fig. 10 | Photograph of the evaporative layer surface after adsorbing moisture and evaporating for 12 hours under one-sun illumination (25 ℃, 70 % RH). A half of the evaporative layer was covered by gobo as a comparison.

Supplementary Note 2.

To explore whether there is salt transfer and release process between the two parts, an assembled device was exposed to environment of 25 °C with 70% RH, and one-sun illumination was applied on a half of the evaporative layer. After 12 hours of persistent intense moisture adsorption and water evaporation, there is no obvious salt formation on the surface of the whole evaporative layer, indicating that LiCl cannot be massively consumed and crystallized at the evaporative layer during operating process.

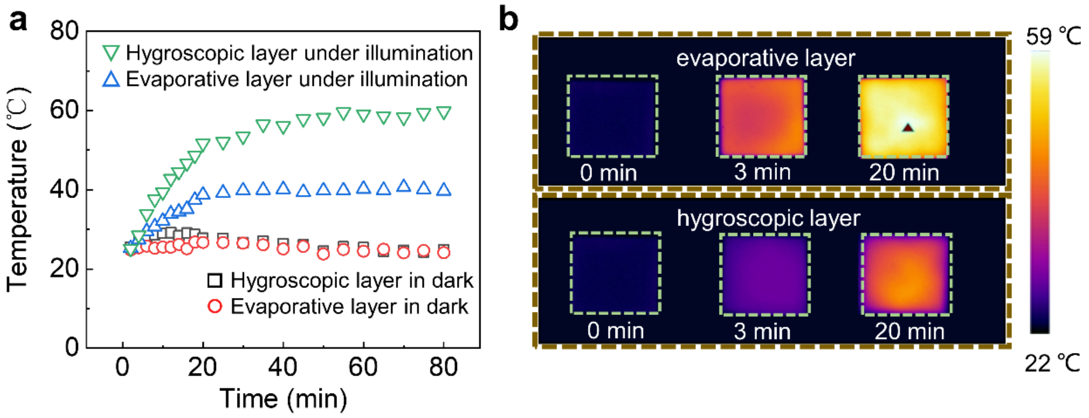

Supplementary Fig. 11 | Heat transfer process between the hygroscopic layer and the evaporative later. (a) Temperature evolution of the hydroscopic layer and the evaporative layer under one-sun illumination and dark conditions. The temperature and relative humidity of the environment are 25 ℃ and 60%. (b) IR thermal images of the hygroscopic layer and the evaporative layer under one-sun illumination after a fixed time.

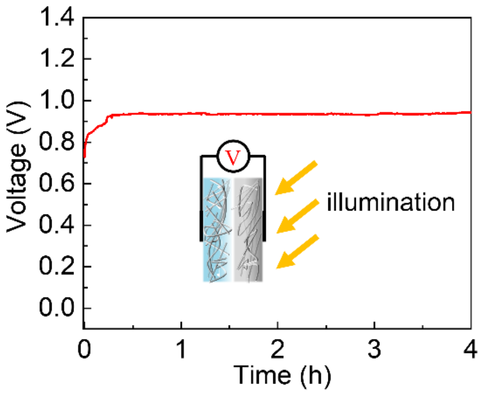

Supplementary Fig. 12 | Voltage output of the SSEG under one-sun illumination. The temperature and relative humidity of the environment are 25 ℃ and 60%.

Supplementary Note 3.

To evaluate the heat transfer process between the two layers, two assembled devices were placed in an environment of 25 ℃ and 60% RH, while illumination of one sun was applied to one of the devices. The surface temperature of each layer of the two devices was measured and recorded simultaneously. As shown in Supplementary Fig. 11a, temperature of the hygroscopic layer surface in dark presents an obviously higher level than that of the evaporative layer surface during the first 20 minutes. The exothermic behavior caused by rapid deliquescence of lithium chloride in the hygroscopic layer is responsible for the temperature difference, which gradually diminishes and disappears because of heat transfer from the hygroscopic layer to the evaporative layer and air. The direction of heat transfer between the two layers under illumination is opposite. The surface temperature of the evaporative layer is much higher than that of the hygroscopic layer due to its photothermal characteristic (Supplementary Fig. 11b), resulting in a persistent heat transfer from the evaporative layer to the hygroscopic layer. When the device was under one-sun illumination, an enhanced voltage output of about 0.9 V was obtained as shown in Supplementary Fig. 12, indicating that the heat transfer will not adversely affect the electricity generation.

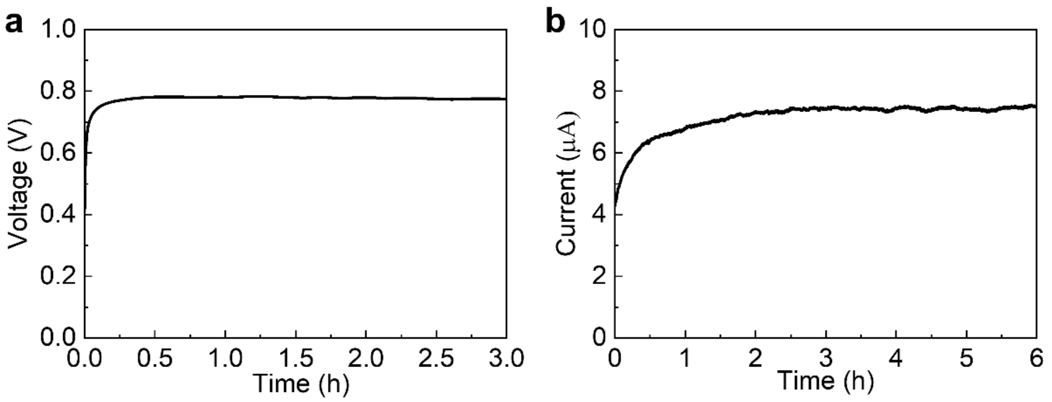

Supplementary Fig. 13 | Electric outputs of the SSEG with integration of moisture adsorption and evaporation. (a) The open-circuit voltage and (b) The short-circuit current delivered by a device without encapsulation. Both of these two signals were measured in an ambient environment of 25 ℃, 60% RH.

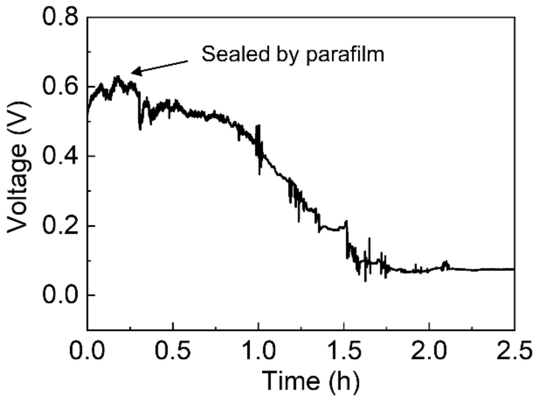

Supplementary Fig. 14 | Voltage output of the SSEG in a closed system. The device operated normally and then was wrapped completely with parafilm.

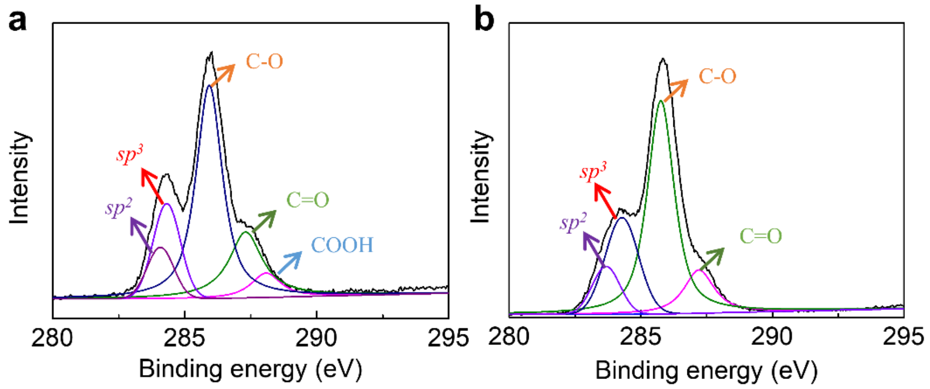

Supplementary Fig. 15 | Chemical characterization of the SSEG. C_{1s} X-ray photoelectron spectroscopy (XPS) results of (a) LiCl-loaded and (b) carbon-black-loaded cellulon paper.

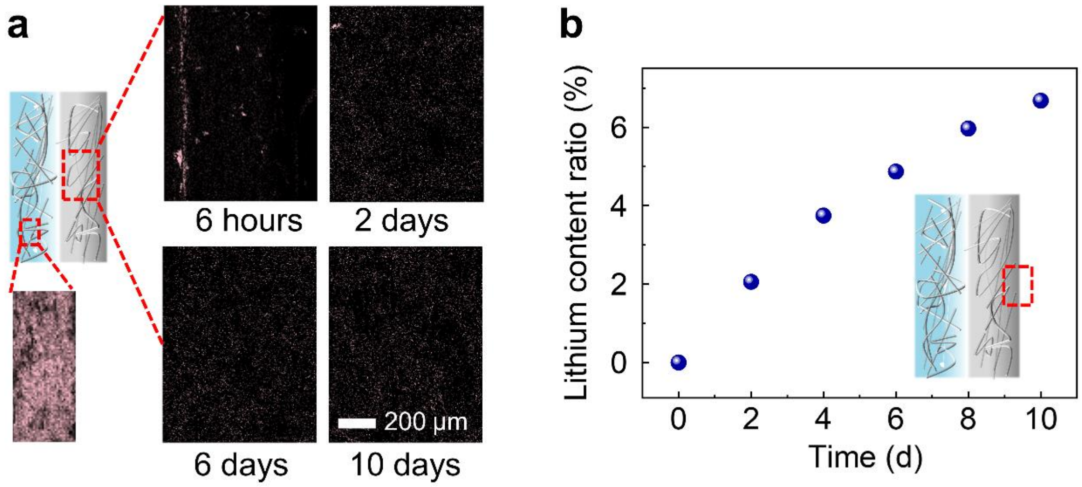

Supplementary Fig. 16 | Transportation of ions during the power generation. (a) Element mapping images of energy dispersive spectrum of chlorine in the evaporative layer during ten days. (b) Evolution of lithium content ratio within the outer surface of the evaporative layer during ten days.

Supplementary Note 4.

Energy dispersive spectrum of chloride in the evaporative layer was tracked to reveal the distribution of lithium chloride in electricity generation. A small amount of chlorine ions diffuses from the hygroscopic layer to the evaporative layer after six hours' operation of the device, and are mainly distributed on the inner side of the evaporative layer. In the next ten days, the chlorine ions gradually diffuse and distribute evenly across the evaporative layer, while the chlorine content is little changed. The results are consistent with the presumption that the transfer of chlorine ions is rather retarded and even negligible because of the ion selectivity of the negatively charged cellulon channels. To confirm the transfer process of lithium ions, x-ray photoelectron spectroscopy of the evaporative layer outside surface was measured as shown in Supplementary Fig. 16b. The lithium content ratio within the outer surface of the evaporative layer increases steadily from 0% to 6.68% during 10 days, indicating that lithium ions slowly flow from the hygroscopic layer to the evaporative layer, which coherently imply that positive ions dominate the total diffused ions.

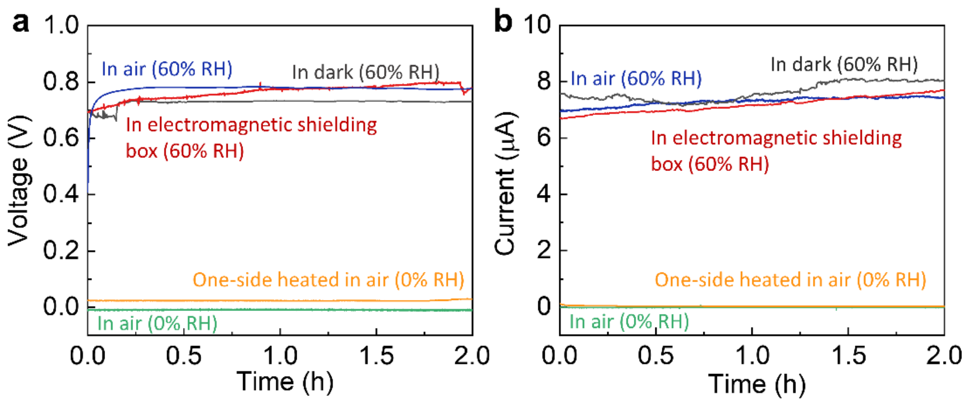

Supplementary Fig. 17 | Electricity generation performance of the SSEG under different environment conditions. (a)Voltage and (b) current output of the SSEG under different environment conditions. A heat plate of 50 ℃ was attached to the surface of hygroscopic layer to evaluate the contribution of thermoelectric effect between the two layers. Other tests were carried out at room temperature.

Supplementary Note 5.

SSEG maintains high electric outputs without any illumination or electromagnetic radiation input at 60% RH, suggesting that neither light nor electromagnetic radiation can be the energy source. The contribution of thermoelectric is further excluded by the near-zero electricity generation from the device with one-side heated at 0% RH. Only when the SSEG is exposed to the air with both moisture adsorption and water evaporation, electricity could be generated rapidly and sustained for a long term. Otherwise, the electric output is significantly degraded, indicating that electricity generation is closely related to the moisture adsorption and water evaporation through the device. As other energy sources have been strictly ruled out, the chemical potential changes of water phase transition during the moisture adsorption, [1,2] and the ambient thermal energy absorbed by natural evaporation are reasonably considered as the main energy source. [3]

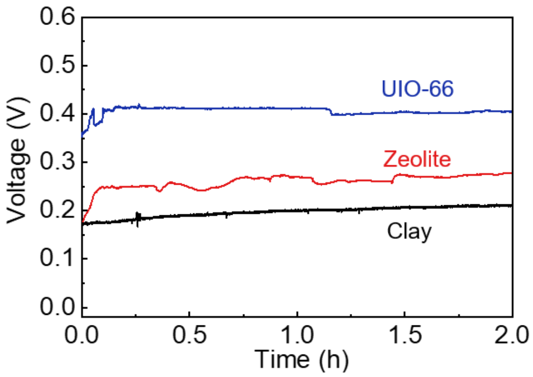

Supplementary Fig. 18 | Voltage output of the SSEGs with non-ionic hygroscopic agents. The experiments were carried out at 25 ℃ and 60 % RH.

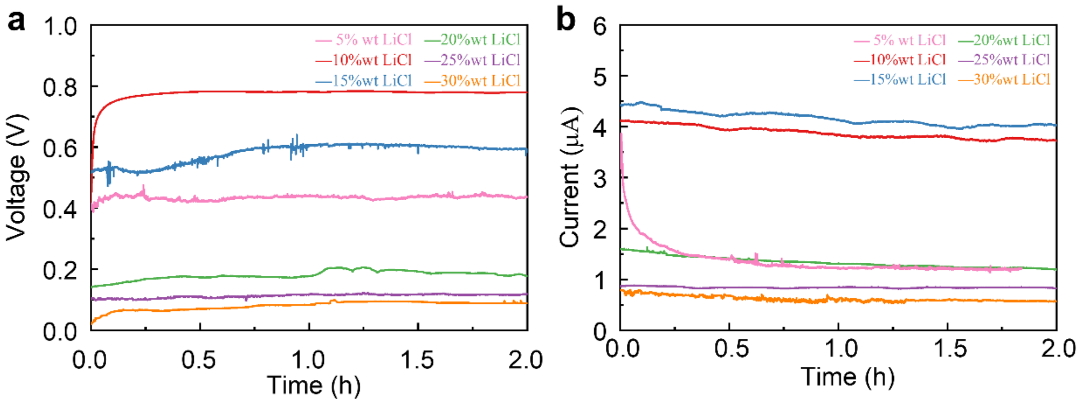

Supplementary Fig. 19 | Electric outputs of the device with different content of hygroscopic agents. (a) The open-circuit voltage and (b) The short-circuit current delivered by devices with different content of LiCl in hygroscopic layer. The testing was performed in the ambient environment at 55-65% RH.

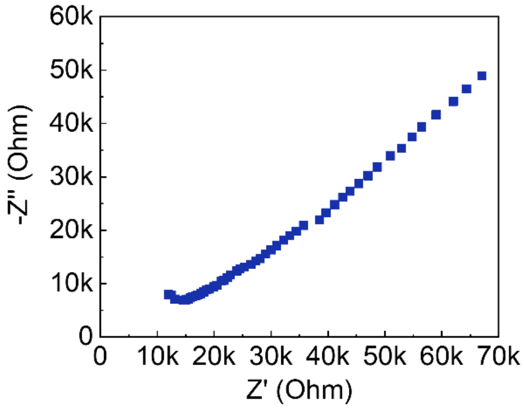

Supplementary Fig. 20 | The electrochemical impedance of the SSEG at 50% RH.

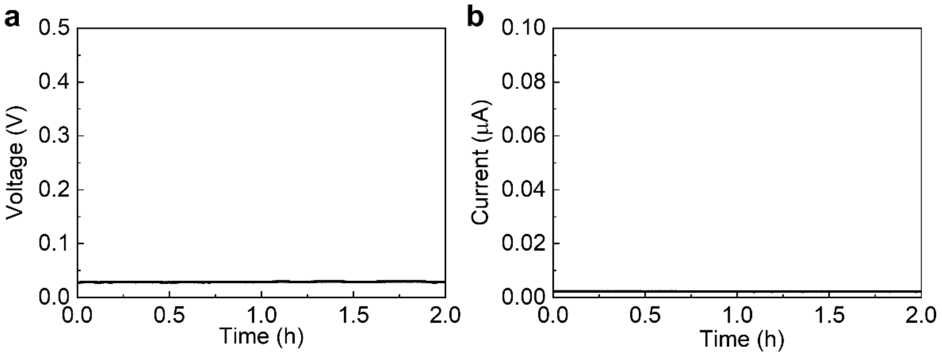

Supplementary Fig. 21 | Electric outputs of the device without doping LiCl. (a) The open-circuit voltage and (b) The short-circuit current delivered by a device without doping LiCl. These two signals were measured in an ambient environment of 25 ℃, 60% RH.

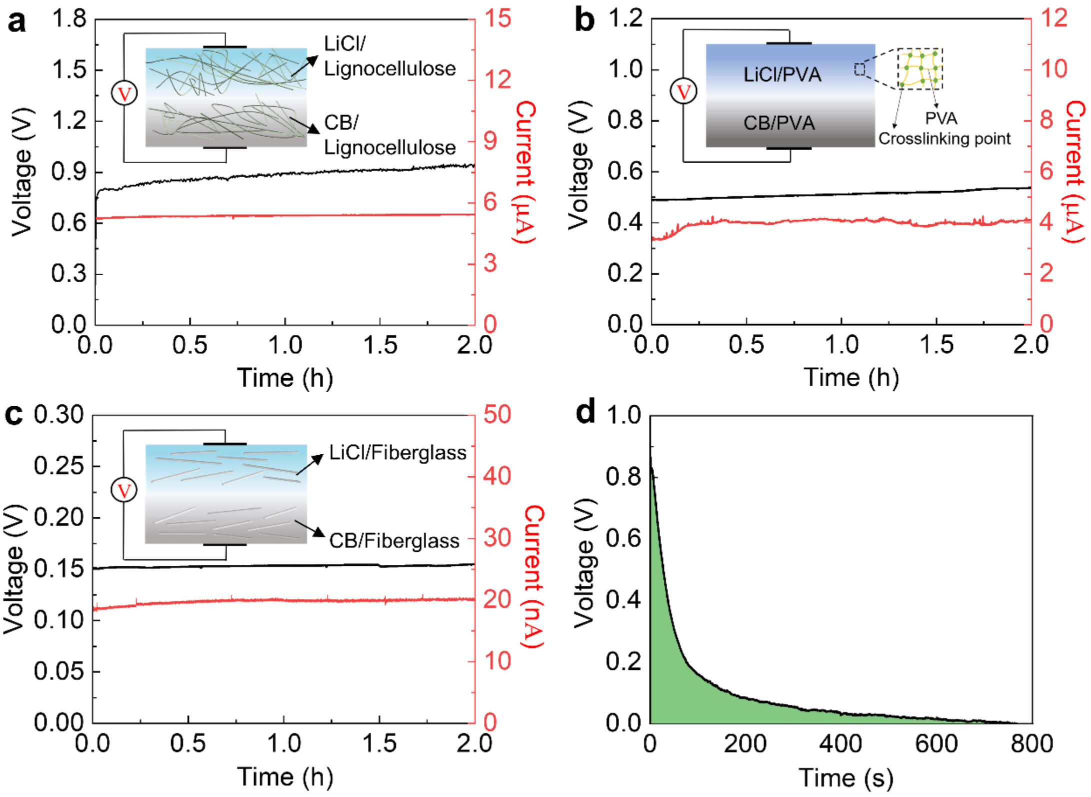

Supplementary Fig. 22 | Electric outputs of controlled devices with different constructing materials and galvanostatic discharge curve of the device. Voltage and current output of the device made from (a) lignocellulose, (b) polyvinyl alcohol hydrogel and (c) fiberglass. The environmental conditions are 25 ℃, 60% RH. (d) The galvanostatic discharge curve of the device made from cellulose aerogel at 23 ℃, 55% RH. The discharge current is 10 μA.

Supplementary Note 6.

Three types of membranes (lignocellulose aerogel, polyvinyl alcohol hydrogel and fiberglass film) were used to replace the cellulon paper for fabricating the devices, and the power generation performance of the obtained devices was accordingly tested. As shown in Supplementary Figs. 22a and b, controlled devices made from lignocellulose aerogel and polyvinyl alcohol (PVA) hydrogel, both with negatively charged channels and abundant hydroxy groups,[4] yielded similar and even higher electric output of 0.9 V, 5.5 μA and 0.5 V, 4 μA, respectively. As the PVA hydrogel contains water inside, the corresponding evaporative layer has a reduced water content gradient between two layers initially, which leads to a slight decrease in the electricity. By contrast, controlled device made from fiberglass film, which feature highly porous structure without functional groups and negatively charged channels, yielded much lower electric output of about 0.15 V, 20 nA (Supplementary Fig. 22c). The significant difference in electric output indicates that the porous structure is not the only requirement for the constructing material, while oxygen-containing functional groups and the negatively charged channels by chemical dissociation that provide free protons and selectivity to the opposite ions, play a crucial role in electricity generation.

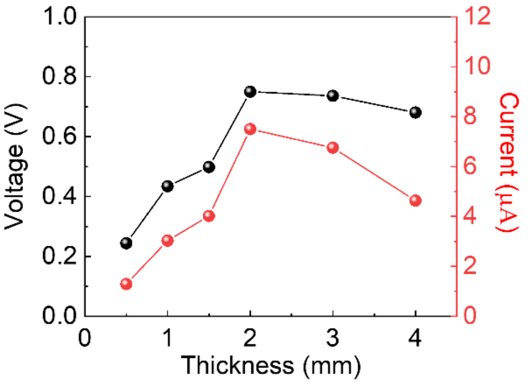

Supplementary Fig. 23 | Voltage and current output of the SSEG with different thickness tested at 25 ℃ and 60% RH.

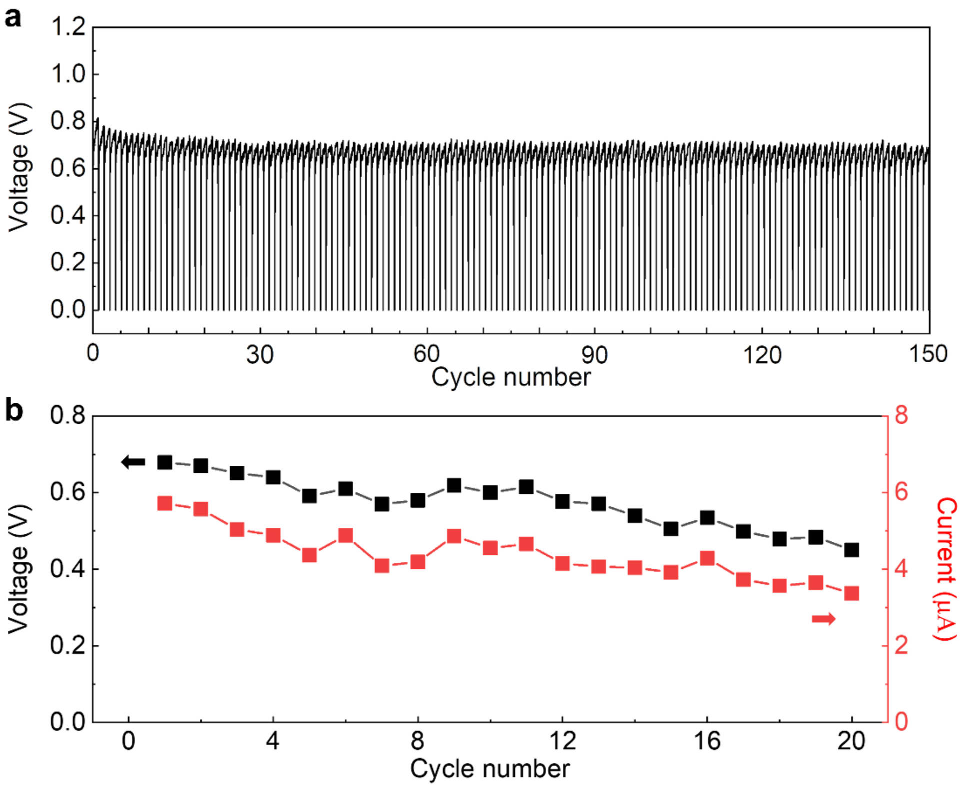

Supplementary Fig. 24丨Cyclic stability of the SSEG. (a) Cyclic performance of periodic autonomous charge-positive discharge for a SSEG. The testing interval of each charge-discharge cycle is 1.5 minutes. (b) Cyclic performance of electricity generation for a SSEG with periodic water adsorption-dehydration. The device was exposed to air until stably generating electricity for 2 hours and then dried at 100 ℃ for 1 hour to completely remove water. The tests were carried out at room temperature with a relative humidity of 40%.

Supplementary Note 7.

The voltage increased from 0.24 V to a maximum of ~0.75 V when the thickness of the device increased from 0.5 to 2 mm and then slightly decreased with further increasing the thickness to 4 mm. This is because the device thickness will affect the ion diffusion and the distribution of water content gradient. A SSEG with a thickness of 2 mm yielded an optimum voltage and current output of 0.78 V and 7.5 μA, respectively.

The voltage output of the SSEG could rapidly and spontaneously recover after discharging. As shown in Supplementary Fig. 24a, the voltage of the device is resumed spontaneously after the discharging process and maintains a retention of 85% after 150 cycles. The high cyclic stability of the device could be attributed to the strong moisture capture ability of the hygroscopic layer and the rapid water flow driven by the evaporation process. The cyclic stability of electricity performance for a SSEG with periodic water adsorption-dehydration is also tested and demonstrated in Supplementary Fig. 24b. The generated electricity of the device decays slowly with increasing the cyclic number due to the surface passivation of LiCl during the water adsorption-dehydration process, but it remains a retention of 88% and 67% after 10 and 20 cycles, respectively.[5]

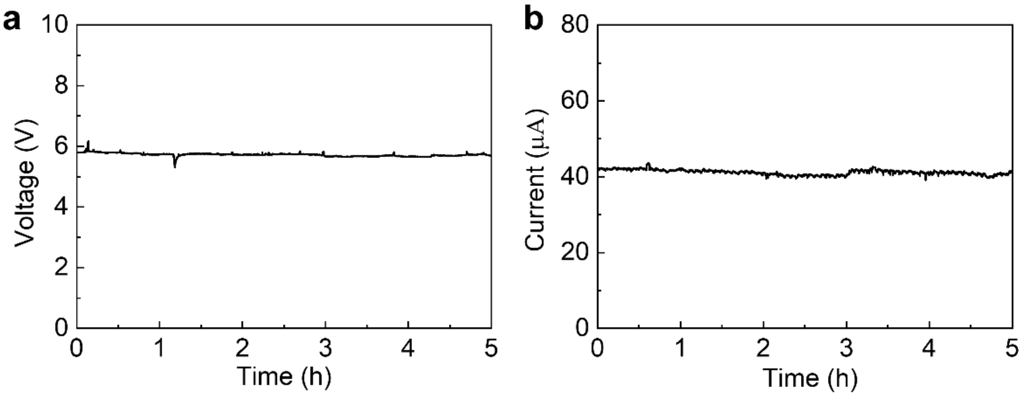

Supplementary Fig. 25 | Electric outputs of integrated devices. (a) The open-circuit voltage and (b) The short-circuit current delivered by 100 SSEGs with series of parallel connections. Both of these two signals were measured in an ambient environment of 23 ℃, 67% RH.

Supplementary Movie 1 Persistent lighting LEDs directly driven by the integrated SSEGs.

Supplementary Movie 2 A cell phone charging by the integrated SSEGs with energy-storage devices.

Supplementary Movie 3 Bluetooth devices powered by the integrated SSEGs with energy-storage devices.

Supplementary Reference

1 Wang, H. et al. Bilayer of polyelectrolyte films for spontaneous power generation in air up to an integrated 1,000 V output. *Nat. Nanotechnol.* **16**, 811–819 (2021).

2 Huang, Y. et al. All-region-applicable, continuous power supply of graphene oxide composite. *Energy Environ. Sci.* **12**, 1848-1856 (2019).

3 Xue, G. et al. Water-evaporation-induced electricity with nanostructured carbon materials. *Nat. Nanotechnol.* **12**, 317-321 (2017).

4 Zhao, F. et al. Highly efficient solar vapour generation via hierarchically nanostructured gels. *Nat. Nanotechnol.* **13,** 489-495 (2018).

5 Zhou, X. et al. Atmospheric Water Harvesting: A Review of Material and Structural Designs. *ACS Mater. lett.* **2***,* 671-684 (2020).
