# Supplementary material for: Self-sustained electricity generator driven by the compatible integration of ambient moisture adsorption and evaporation
Source: Nat Commun. 2022 Jun 25;13:3643. doi: 10.1038/s41467-022-31221-7 (PMC9233698; doi:10.1038/s41467-022-31221-7)
Supplement: Supplementary file 3 — Description of Additional Supplementary Files [file 41467_2022_31221_MOESM3_ESM.pdf]

Description of Additional Supplementary Files:

Supplementary Movie 1: Persistent lighting LEDs directly driven by the integrated SSEGs. The integrated SSEGs consist of a hundred devices connected in a series of parallel connections (ten devices are connected in parallel into a group, then ten groups are connected in series).

Supplementary Movie 2: A cell phone charging by the integrated SSEGs with energy-storage devices. Four capacitors with 1000 microfarads were charged for 10 minutes by the integrated SSEGs. Then the charged capacitors were used to charge a cell phone and a charging time of 15 seconds was obtained.

Supplementary Movie 3: Bluetooth devices powered by the integrated SSEGs with energy-storage devices. Four capacitors with 1000 microfarads were charged for 5 minutes by the integrated SSEGs. Then the charged capacitors were used to drive a Bluetooth signal transmitter and a cell phone was used to connect the signal transmitter and received the signal.
